# Patient vs. clinician-collected cervical cytology for screening for CIN: a systematic review and meta-analysis

**DOI:** 10.1016/j.xagr.2025.100575

**Published:** 2025-10-05

**Authors:** Greg Marchand, Daniela Gonzalez Herrera, Brooke Hamilton, McKenna Robinson, Emily Kline, Sarah Mera, Michelle Koshaba, Greenley Jephson, Nidhi Pulicherla, Ali Azadi

**Affiliations:** 1 Marchand Institute for Minimally Invasive Surgery, Mesa, AZ (Marchand, Gonzalez Herrera, Hamilton, Robinson, Kline, Mera, Koshaba, Jephson and Pulicherla); 2 University of Arizona, College of Medicine Phoenix, AZ (Azadi); 3 Creighton University, School of Medicine Phoenix, AZ (Azadi)

**Keywords:** cervical cancer, self-collection, cervical cytology, systematic review, meta-analysis, diagnostic accuracy

## Abstract

**Objective:**

This systematic review and meta-analysis aimed to compare the diagnostic accuracy of self-collected versus clinician-collected cervical cytology for screening for cervical intraepithelial neoplasia (CIN), using cervical biopsy histopathology as the reference standard. The study sought to evaluate self-collection’s potential to enhance cervical cancer screening participation, particularly among under-screened women, by addressing barriers such as discomfort and logistical challenges.

**Data Sources:**

We searched PubMed (MEDLINE), Web of Science, Scopus, and Cochrane databases from their inception to March 1, 2024, to identify relevant studies comparing self-collected and clinician-collected cervical cytology.

**Study Eligibility Criteria:**

Eligible studies included adult women (≥18 years) undergoing both self-collected and clinician-collected cervical cytology (conventional Pap smear or liquid-based cytology), with cervical biopsy as the reference standard. Studies were required to report diagnostic outcomes (e.g., sensitivity, specificity) and include a clinician-collected comparator. Non-English studies, those lacking biopsy as the reference standard, or single-arm studies were excluded.

**Study Appraisal and Synthesis Methods:**

Two independent reviewers screened studies, extracted data, and assessed methodological quality using the QUADAS-2 tool. A diagnostic meta-analysis was conducted with Review Manager 5.4 to calculate pooled sensitivity and specificity with 95% confidence intervals, using both unweighted and weighted methods. Summary receiver operating characteristic (SROC) curves were generated to visualize diagnostic performance.

**Results:**

Five studies were included, revealing that self-collected cytology had a pooled sensitivity of 0.698 and specificity of 0.805, while clinician-collected cytology showed a higher sensitivity of 0.765 but lower specificity of 0.613, with variation in sensitivity and specificity observed across different self-collection methods (e.g., brushes, tampons, lavages). QUADAS-2 assessment identified methodological concerns, particularly in patient selection and flow and timing, suggesting risks of bias and limited generalizability to primary screening settings. Methodological limitations in studies, including wide variation in self-collection methods, highlight the need for further research.

**Conclusions:**

Self-collected cervical cytology demonstrates reasonable diagnostic accuracy, offering a promising approach to increase screening uptake among under-screened populations by overcoming barriers to traditional clinician-based methods. However, its lower sensitivity compared to clinician-collected cytology underscores the need for careful implementation, including optimized assay selection and targeted population strategies. Heterogeneity in self-collection methods, which varied widely (e.g., Mermaid rinse, tampons, brushes), may contribute to differences in diagnostic performance and underscores the need for standardized approaches. Further high-quality research is essential to refine self-collection techniques and strengthen its role in global cervical cancer prevention efforts.

**AJOG MFM at a Glance**

**A. Why was this study conducted?**

 • To compare diagnostic accuracy of self-collected versus clinician-collected cervical cytology for screening for cervical intraepithelial neoplasia (CIN), using biopsy as the reference standard.

 • To address screening barriers and evaluate self-collection’s potential to increase participation in cervical cancer prevention.

**B. What are the key findings?**

 • Self-collected cytology had a pooled sensitivity of 0.698 and specificity of 0.805; clinician-collected had higher sensitivity (0.765) but lower specificity (0.613).

 • Methodological limitations in studies highlight the need for further research.

**C. What does this study add to what is already known?**

 • Provides the first meta-analysis comparing self- and clinician-collected cytology accuracy, informing self-sampling’s role in cervical cancer screening programs.

 • Supports self-collection as a viable option to improve screening uptake, especially for under-screened women.

## Introduction

Cervical cancer remains a significant global health challenge, ranking as the fourth most common cancer and the fourth leading cause of cancer mortality among women worldwide.^1^ Clinician-collected Papanicolaou (Pap) smear screening programs have proven effective for the prevention and early detection of cervical cancer, yet their impact is constrained by low participation rates.^2^ Women who do not engage in regular screening are at the highest risk of developing cervical cancer, underscoring the critical issue of screening non-attendance.^3^^,^^4^

Barriers to clinician-collected Pap smears include embarrassment, discomfort associated with invasive pelvic examinations, logistical inconveniences, and, in smaller communities, familiarity with healthcare providers, all of which deter participation.^5^, ^6^, ^7^, ^8^

The U.S. Preventive Services Task Force (USPSTF) has recently addressed these barriers in its 2024 draft recommendation statement, which, for the first time, endorses self-collection of vaginal samples for high-risk human papillomavirus (hrHPV) testing as an acceptable screening method for women aged 30 to 65.^1^ This landmark update recognizes self-collection’s potential to increase screening uptake, particularly among under-screened populations, by offering a less invasive and more accessible alternative to clinician-collected samples. The USPSTF’s recommendation highlights the accuracy of self-collected hrHPV testing and its ability to overcome cultural, emotional, and logistical barriers, aligning with efforts to reduce cervical cancer disparities, especially among Black, Hispanic, and Native American women who face higher incidence and mortality rates.^1^ While the USPSTF’s focus is on hrHPV testing, this development underscores the growing interest in self-sampling methods, including their potential application to cervical cytology, which is the focus of this study.

Self-collection of cervicovaginal samples offers a promising strategy to enhance screening participation, particularly for under-screened or never-screened women, who are most vulnerable to cervical cancer.^4^^,^^9^, ^10^, ^11^, ^12^, ^13^, ^14^, ^15^, ^16^ Research has demonstrated that self-sampling significantly improves uptake, with studies showing its feasibility and acceptance for hrHPV testing.^16^, ^17^, ^18^, ^19^, ^20^, ^21^, ^22^ However, the application of self-collection to cytological analysis, such as Pap smears, is gaining attention, particularly for triaging positive HPV self-tests or as a standalone screening method.^14^^,^^15^ In light of the USPSTF’s endorsement of self-collected hrHPV testing, evaluating the diagnostic accuracy of self-collected cytology becomes increasingly relevant, as it could complement HPV-based screening strategies and further expand access to cervical cancer prevention.

Despite this potential, the diagnostic accuracy of self-collected cytology compared to clinician-collected cytology remains uncertain, with limited and inconsistent direct comparisons using cervical biopsy histopathology as the reference standard. For instance, Singla et al. reported a sensitivity of 64.7% and specificity of 86.4% for self-collected cytology versus 47.1% and 81.0% for clinician-collected cytology.^2^ Jones et al. found 75% sensitivity and 80% specificity for self-collection compared to 88% and 58% for clinician-collection.^23^ Othman et al. noted moderate agreement (κ = 0.57) between the 2 methods.^24^ These variable findings highlight a critical evidence gap that our study seeks to address.

This systematic review and meta-analysis is particularly timely given the USPSTF’s 2024 draft recommendation, as it evaluates the diagnostic accuracy of self-collected versus clinician-collected cervical cytology for screening for CIN, using histopathology from cervical biopsy as the reference standard. By providing a comprehensive analysis of self-collected cytology’s performance, our study contributes to the broader discourse on self-sampling innovations, offering insights that could inform the integration of cytology-based self-collection into screening programs alongside or following hrHPV testing. Such advancements are essential for optimizing cervical cancer prevention strategies and reducing global health disparities.

## Methods

This systematic review and meta-analysis adhered to the Preferred Reporting Items for Systematic Reviews and Meta-Analyses (PRISMA) guidelines.^25^

### Eligibility criteria

Studies were eligible if they: (1) included adult women (≥18 years) undergoing self-collected cervical cytology (conventional Pap smear or liquid-based cytology); (2) included a comparator group undergoing clinician-collected cervical cytology (conventional Pap smear or liquid-based cytology); (3) reported at least 1 diagnostic outcome measure (e.g., sensitivity, specificity); and (4) used cervical biopsy as the reference standard for confirming CIN. Studies were excluded if they: (1) were not in English; (2) lacked a clinician-collected comparator; (3) used a reference standard other than cervical biopsy; or (4) were conference abstracts, case reports, case series, editorials, commentaries, letters, or reviews.

### Search strategy

We searched Web of Science, PubMed (MEDLINE), Scopus, and Cochrane databases from inception to March 1, 2024, using the terms: ("pap smear" OR "pap smears" OR "Pap test" OR "pap tests" OR "Papanicolaou smear" OR "Papanicolaou smears" OR "Papanicolaou test" OR "Papanicolaou tests" OR "cervical smear" OR "cervical smears" OR "cervical cytology") AND ("self" OR "self-test" OR "self-testing" OR "home-based test" OR "home-based testing" OR "home test" OR "home testing" OR "clinic-based test" OR "clinic-based testing" OR "community-based test" OR "pharmacy-based test" OR "self-administer" OR "self-sampling" OR "self-collecting" OR "self-collected" OR "self-collection" OR "self- versus provider-collected" OR "self- and provider-collected" OR "self-versus physician-collected" OR "self- and physician-collected").

### Study selection

Studies were managed in EndNote X9 and exported to Microsoft Excel for screening. We used a 2-step process: (1) title and abstract screening, followed by (2) full-text review of potentially eligible articles. Two independent reviewers performed each step, resolving discrepancies through discussion or consultation with a third senior reviewer.

### Data extraction

Two reviewers independently extracted data into a standardized Microsoft Excel form, including author, year, country, study design, setting, sample size, participant age, reason for testing, self-collection device, sampling site, preservation medium, and group sizes. For the cervical biopsy reference standard, we extracted procedure details, histopathological criteria, and positive case counts. True positives (TP), true negatives (TN), false positives (FP), and false negatives (FN) were recorded for both cytology methods. Discrepancies were resolved by consensus.

### Quality assessment

We evaluated study quality using the QUADAS-2 tool, which assesses risk of bias and applicability across 4 domains: patient selection, index test, reference standard, and flow and timing.^26^

### Statistical analysis

A diagnostic meta-analysis compared the accuracy of self-collected and clinician-collected cytology. Pooled sensitivity and specificity, with 95% confidence intervals (CIs), were calculated using unweighted (simple averaging) and weighted (sample size-adjusted) methods. Summary receiver operating characteristic (SROC) curves visualized sensitivity-specificity relationships. Analyses were performed in Review Manager (RevMan) 5.4.

## Results

### Study selection

**Figure 1:**
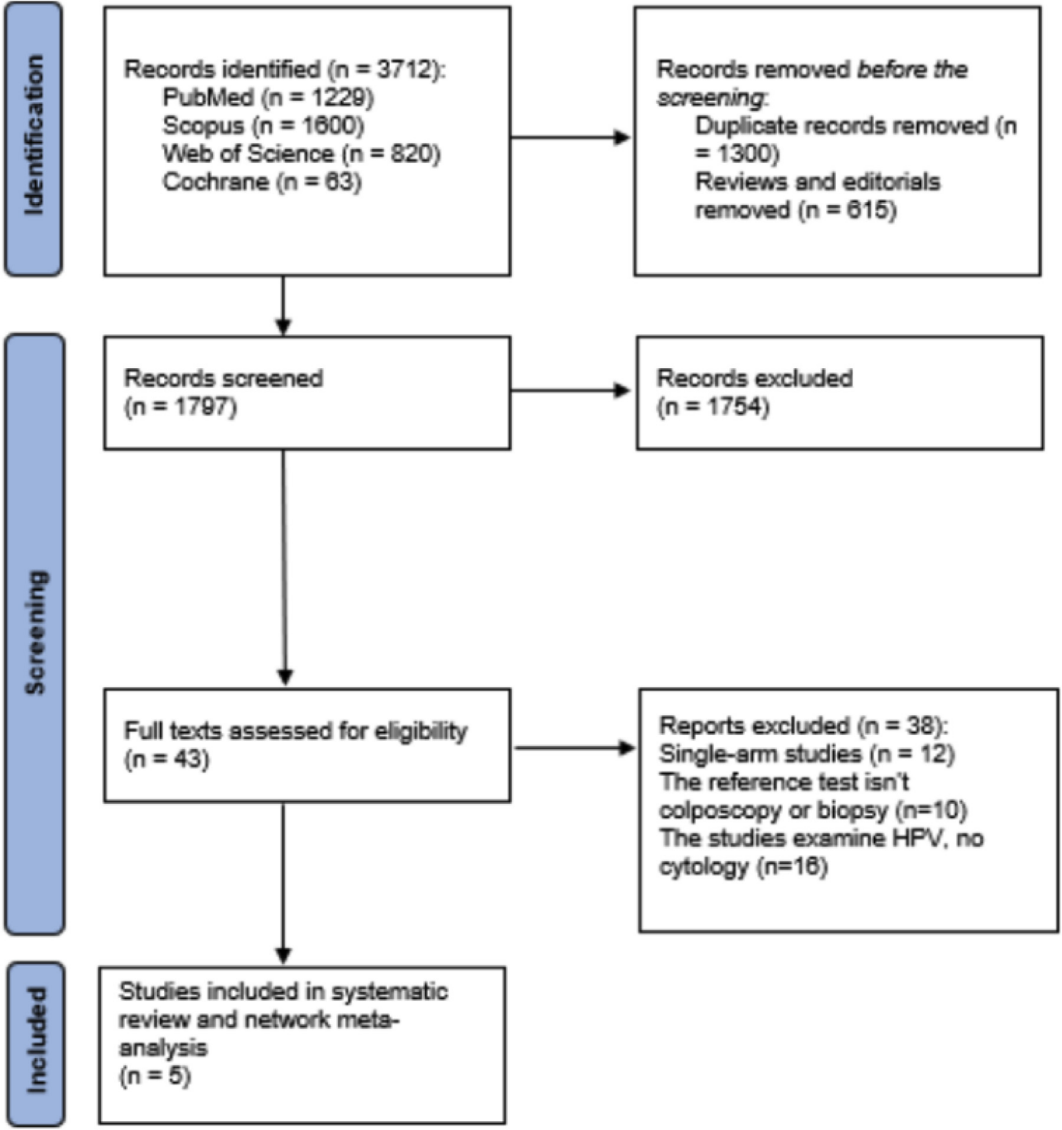
PRISMA Flow Diagram of Study Selection.

The study selection process is outlined in the PRISMA flow diagram (Figure 1). Initial database searches yielded 3712 records from PubMed (Medline) (n = 1229), Scopus (n = 1600), Web of Science (n = 820), and Cochrane (n = 63). After removing 1300 duplicate records and 615 reviews and editorials, 1797 records were screened based on titles and abstracts, resulting in the exclusion of 1754 records. The full texts of 43 articles were assessed for eligibility, and 38 were subsequently excluded because they were single-arm studies (n = 12), did not use colposcopy or biopsy as a reference test (n = 10), or examined HPV without cytology (n = 16). Ultimately, 5 studies met all inclusion criteria and were included in the systematic review and meta-analysis.

Marchand. Patient vs. clinician-collected cervical cytology for screening for CIN. Am J Obstet Gynecol 2025

### Quality assessment

**Figure 2:**
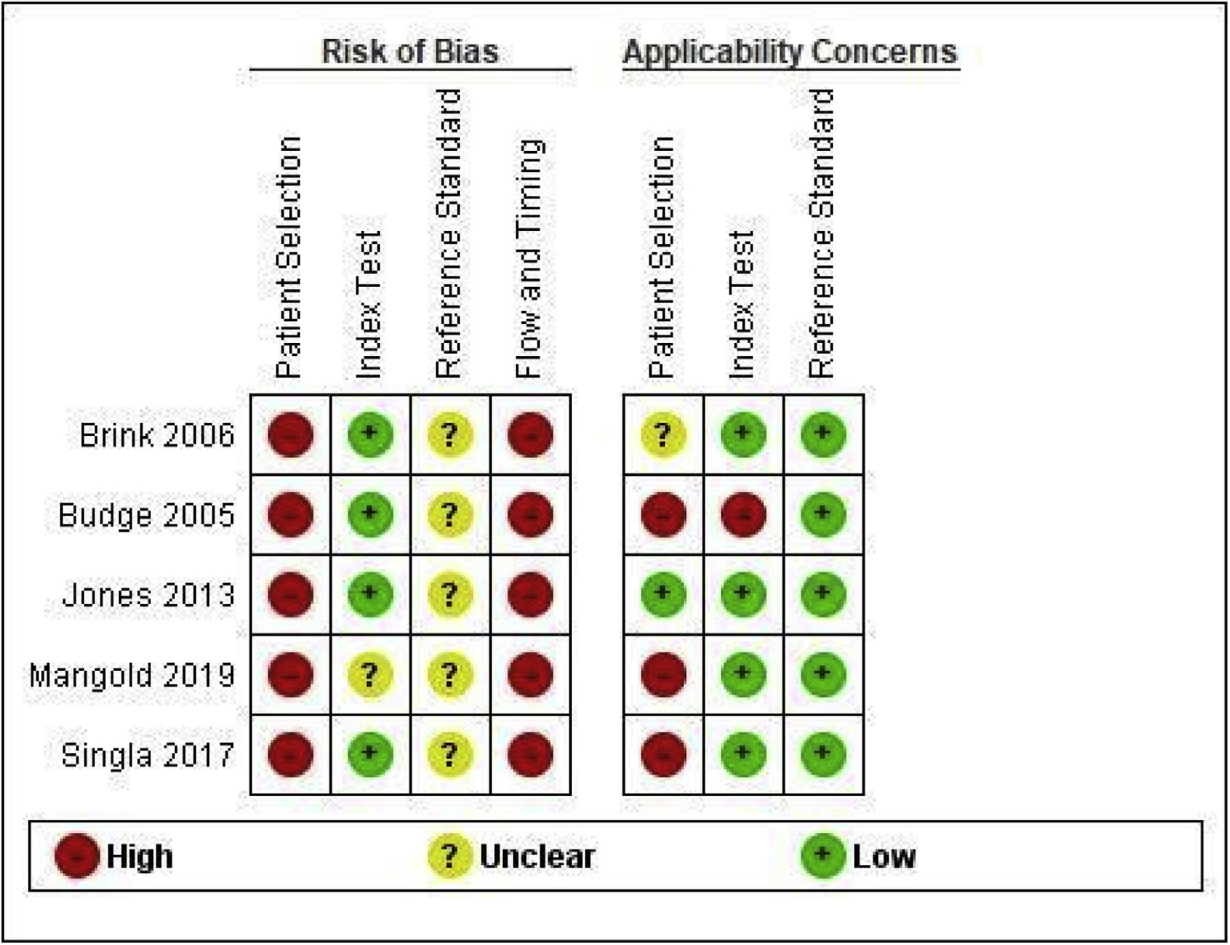
QUADAS-2 Risk of Bias Graph for Included Studies.

**Figure 3:**
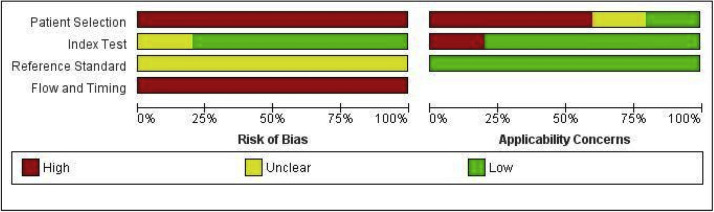
QUADAS-2 Summary of Bias and Applicability for Included Studies.

The methodological quality of the 5 included studies was assessed using the QUADAS-2 tool (Figure 2, Figure 3). Significant methodological concerns were identified, particularly regarding risk of bias. All studies were rated at high risk of bias for Patient Selection, primarily due to recruitment from high-risk referral populations, and for Flow and Timing, mostly due to partial verification bias or inappropriate test intervals. Furthermore, the risk of bias related to the Reference Standard was consistently unclear across studies, often due to inadequate reporting of blinding. Applicability concerns were also high for Patient Selection in most studies, limiting generalizability to primary screening settings.

Marchand. Patient vs. clinician-collected cervical cytology for screening for CIN. Am J Obstet Gynecol 2025

Marchand. Patient vs. clinician-collected cervical cytology for screening for CIN. Am J Obstet Gynecol 2025

### Study characteristics

Study Characteristics: Table 1 summarizes the included studies’ key features. Originating from the Netherlands, Australia, USA, and Germany, most used comparative diagnostic accuracy designs, except Budge (2005), which was observational. Settings included gynecological outpatient departments, ambulatory clinics, sexual health clinics, and colposcopy clinics. Sample sizes ranged from 39 to 209, with mean ages of 33.1–42.7 years where reported. Participants were involved due to abnormal cytology referrals (e.g., moderate dysplasia, equivocal Pap smears), screening clinic attendance, or colposcopy consultations.

**Table 1:** Summary of key characteristics of the included studies

Author	Country	Study design	Setting	Total sample size	Mean Age (SD)	Reason for Current Test/Visit (e.g., Routine screening, Colposcopy clinic attendees, non-responders)
Brink et al.^27^	Netherlands	Cross-sectional diagnostic accuracy	Gynecological outpatient departments	96	35.4 (8.3)	Referral for moderate dysplasia or worse, or repeat equivocal Pap smear (n=64); Healthy volunteers (n=32)
Budge et al.^28^	Australia	Comparative observational study	Colposcopy clinic & Sexual health clinics	209	NR	Colposcopy clinic attendees & Sexual health clinic attendees
Jones et al.^23^	USA	Comparative diagnostic accuracy	Three ambulatory clinics	195*	33.1 (16.4)	Attending clinics for cervical cancer screening
Mangold et al.^29^	Germany	Comparative diagnostic accuracy	Colposcopy clinic	208	NR	Colposcopy clinic attendees
Singla et al.^30^	Australia	Prospective diagnostic accuracy	Colposcopy Outpatients Clinic	39	42.7 (37.7)	Colposcopic consultation attendees

NR, Not Reported; SD, Standard Deviation.

⁎ Only 167 included in the sensitivity and specificity analysis

### Sample collection methods

Table 2 details self- and clinician-sampling methods. Self-sampling used devices like the Mermaid (upper vagina/cervix rinse), Johnson & Johnson tampons (vagina), Delphi Screener (vaginal/cervical lavage), Rovers® Viba-Brush (vagina), and Cytobrooms (cervix), preserved in SurePath or ThinPrep media. Clinician-sampling used endocervical brushes, conventional Pap smears, liquid-based cytology, Rovers® Cervex-Brushes, and Cervibrooms, preserved similarly.

**Table 2:** Summary of the sampling data and sample size in each study group

Study	Self-sampling group			Physician-sampling group		
	Device, site of the sample	Medium	Sample size	Device, site of the sample	Medium	Sample
Brink et al.^27^	Mermaid cervicovaginal self-sampling device,rinses upper vagina/cervix	SurePath preservation solution	96	Endocervical brush, cervix	SurePath preservation solution (LBC)	95
Budge et al.^28^	Johnson & Johnson tampon, vagina	ThinPrep PreservCyt fluid	209	Conventional Pap smear, cervix	Conventional smear slide preparation	209
Jones et al.^23^	Delphi Screener, vaginal/cervical lavage	ThinPrep PreservCyt fluid	195	Clinician-collected Liquid Based Cytology, cervix	ThinPrep PreservCyt fluid	195*
Mangold et al.^29^	Rovers® Viba-Brush (swab), vagina	ThinPrep PreservCyt fluid	208	Rovers® Cervex-Brush, cervix	ThinPrep PreservCyt fluid	208
Singla et al.^30^	Cytobroom, self-inserted to feel cervix	ThinPrep jar	39	Cervibroom, cervix	ThinPrep jar	39

⁎ Only 167 included in the sensitivity and specificity analysis.

### Reference standards

Reference standards included colposcopy and biopsy with thresholds such as ≥CIN2, ≥CIN3, or ≥CIN1. Most used colposcopy and biopsy with a ≥CIN2 threshold. One study included ≥CIN2 or high-grade epithelial abnormality (CIN 1-3 or HGEA) via colposcopy/biopsy or endocervical curettage; others used ≥CIN3 or ≥CIN1.

### Outcomes

**Figure 4:**
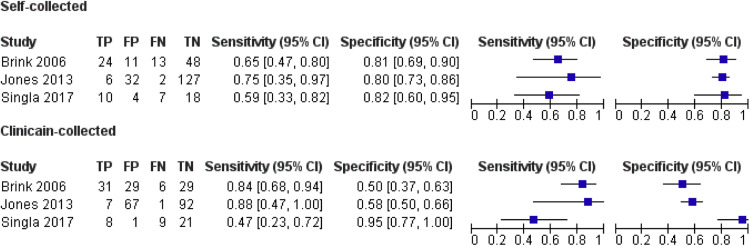
Forest Plot of Sensitivity and Specificity Across Studies.

**Figure 5:**
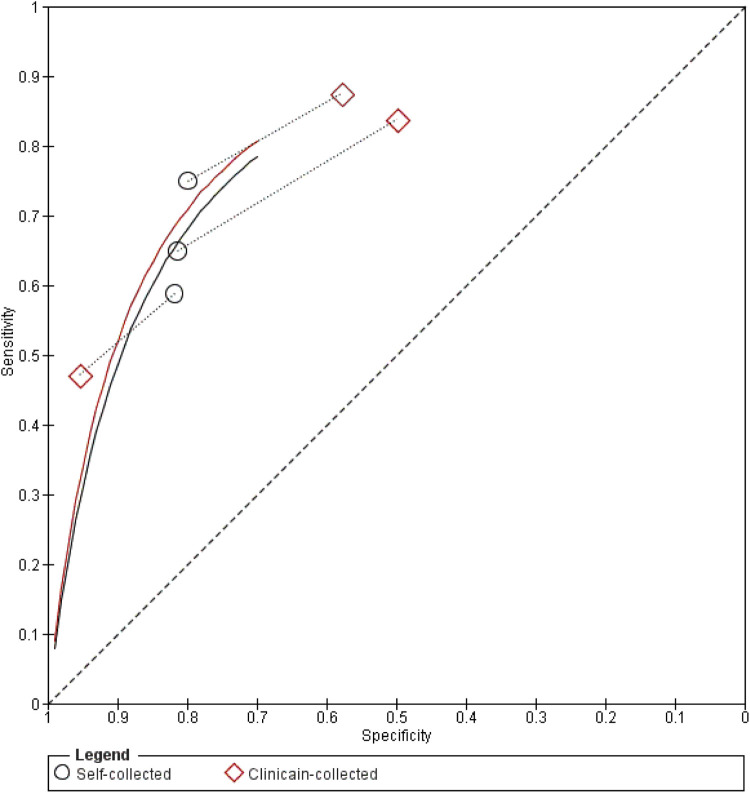
SROC Graph of Sensitivity and Specificity by Test Method.

Self-collected cytology had a pooled sensitivity of 0.698 and specificity of 0.805; clinician-collected had 0.765 and 0.613, respectively, with variation in sensitivity and specificity observed across different self-collection methods (e.g., brushes, tampons, lavages). Figure 4 shows study-specific results, and Figure 5 (SROC graph) compares both methods visually. Two studies (Budge 2005, Mangold 2019) were excluded from the meta-analysis due to insufficient data. Budge (2005) reported 17% sensitivity and 98% specificity for self-collected versus 74% and 95% for clinician-collected (≥CIN2/HGEA cutoff).^28^ Mangold (2019) reported 45% sensitivity and 91% specificity for self-collected, with 21/47 true positives and 43/47 true negatives for clinician-collected (≥CIN3 cutoff).^29^ Variability was evident across studies.

Marchand. Patient vs. clinician-collected cervical cytology for screening for CIN. Am J Obstet Gynecol 2025

Marchand. Patient vs. clinician-collected cervical cytology for screening for CIN. Am J Obstet Gynecol 2025

## Discussion

This systematic review and meta-analysis compares self-collected and clinician-collected cervical cytology for CIN screening, outlining the benefits and limitations of self-sampling. Self-collected cytology showed a pooled sensitivity of 0.698 and specificity of 0.805, while clinician-collected cytology had higher sensitivity (0.765) but lower specificity (0.613). This suggests self-collection reduces false positives but may miss some cases, aligning with variable results from other studies. Singla et al. reported 64.7% sensitivity and 86.4% specificity for self-collection versus 47.1% and 81.0% for clinician-collection.^30^ Jones et al. found 75% sensitivity and 80% specificity for self-collection versus 88% and 58% for clinician-collection.^23^

Cho et al., focusing on urinary HPV testing, reported a relative sensitivity of 0.84 (95% CI 0.78–0.91) for self-collected samples, with lower specificity than clinician-collected samples. In colposcopy clinics, self-collected cytology had 79% sensitivity versus 93% for clinician-collected smears, with comparable specificities (48%, 95% CI 0.42–0.54 vs. 42%, 95% CI 0.36–0.48).^31^ These findings highlight differing performance traits between methods, impacting CIN detection. Cho et al. also noted better self-sampling results in low- and middle-income countries, possibly due to higher HPV prevalence.^32^^,^^33^

Arbyn et al.’s meta-analysis on hrHPV testing showed PCR-based assays had equal sensitivity for self- and clinician-samples (pooled ratio 0.99, 95% CI 0.97–1.02), unlike signal amplification assays (pooled ratio 0.85, 95% CI 0.80–0.89).^34^ They cautioned that cytology’s lower accuracy may require further study and suggested assessing testers’ social environments at home.

Self-sampling suits under-screened women or those with clinic access issues, ideally tested in small, controlled areas first for cost-effectiveness and triage.^35^

To our knowledge, this is the first diagnostic meta-analysis comparing self-collected and clinician-collected cytology accuracy. PRISMA adherence strengthens our evidence, but limitations include methodological heterogeneity (patient selection, verification bias per QUADAS-2) and few biopsy-based comparison studies, reducing statistical power and necessitating more research. Additional limitations include the wide variation in self-collection methods across studies, which may affect comparability and generalizability, as the abstract might otherwise imply uniformity in approaches.

Future studies should address these gaps, exploring new devices, preservation methods, and cytological markers, and conducting large randomized trials to assess self-sampling’s effectiveness and cost in diverse populations. Research should compare assay/device/media combinations across settings and patient traits, and evaluate self-sampling’s feasibility for underserved or non-attending women. Categorizing patients before distributing kits could optimize funding, given higher participation in some settings.

## Conclusion

Self-collected cervical cytology has the potential to boost screening and curb cervical cancer, but its diagnostic accuracy lags behind clinician-collected methods. Implementation requires balancing sensitivity-specificity trade-offs, assay availability, and population needs. More research is vital to refine self-collected cytology and enhance global cervical cancer prevention.

## Ethics approval and consent to participate

This Manuscript has been reviewed by the institutional IRB board at Marchand Institute and was found to be exempt from IRB review. (November 2024). Data used was exempt from consent to participate or publish secondary to the nature of the study being a systematic review, retrospectively looking at previously published data.

## Consent to publish

Data used was exempt from consent to participate or publish secondary to the nature of the study being a systematic review, retrospectively looking at previously published data.

## Commitment to diversity

The Marchand Institute remains committed to diversity and tolerance in its research and actively maintains a workplace free of racism and sexism. Greater than half of the authors for this study are female, and many represent diverse backgrounds and under-represented ethnic groups.

## Patient consent

Not applicable to systematic review.

## Prospero Prospective Registration Number

CRD420251029687

## CRediT authorship contribution statement

**Greg Marchand:** Project administration, Data curation, Conceptualization. **Daniela Gonzalez Herrera:** Formal analysis, Data curation. **Brooke Hamilton:** Formal analysis, Data curation, Writing – review & editing. **McKenna Robinson:** Formal analysis, Data curation. **Emily Kline:** Investigation, Data curation. **Sarah Mera:** Formal analysis, Data curation. **Michelle Koshaba:** Formal analysis, Data curation. **Greenley Jephson:** Formal analysis, Data curation. **Nidhi Pulicherla:** Formal analysis, Data curation. **Ali Azadi:** Writing – review & editing, Writing – original draft, Supervision.
